# Integrated transcriptomic and metabolomic analyses reveal heterosis for meat quality of Neijiang pigs

**DOI:** 10.3389/fvets.2024.1493284

**Published:** 2024-11-25

**Authors:** Haifeng Dan, Chengming Liu, Huiling Zhang, Mailin Gan, Yan Wang, Lei Chen, Ye Zhao, Bin Liu, Kangping Zhu, Lili Niu, Li Zhu, Linyuan Shen

**Affiliations:** ^1^Farm Animal Genetic Resources Exploration and Innovation Key Laboratory of Sichuan Province, Sichuan Agricultural University, Chengdu, China; ^2^Key Laboratory of Livestock and Poultry Multi-omics, Ministry of Agriculture and Rural Affairs, College of Animal and Technology, Sichuan Agricultural University, Chengdu, China; ^3^State Key Laboratory of Swine and Poultry Breeding Industry, College of Animal Science and Technology, Sichuan Agricultural University, Chengdu, China; ^4^Sichuan Dekon Livestock Foodstuff Group, Chengdu, China

**Keywords:** pig, carcass characteristic, meat quality, transcriptomics, metabolomics, hybridization

## Abstract

Obese pig breeds have excellent meat quality, while lean pig breeds have high lean meat percentage and feed conversion rate. However, due to their respective shortcomings, obese pig and lean pig breeds are unable to balance production and consumption needs. Therefore, this study crossbred the obese Chinese pig breed Neijiang (NJ) with lean type Large White pigs (LW) to produce Neijiang × Large White(NL) pigs. This study compared the differences in carcass and meat quality traits between NJ pigs and NL pigs, and for the first time comprehensively analyzed the longissimus dorsi muscle of NJ pigs and NL pigs using transcriptomics and metabolomics. The results of slaughter and meat quality testing indicate that the carcass performance of NL pigs was significantly higher than that of NJ pigs, and the excellent meat quality characteristics of NJ pigs were also retained on NL pigs. The results of transcriptomics and metabolomics showed that there were 635 differentially expressed genes (DEGs) and 11 significantly different metabolites (SDM) in the longissimus dorsi muscle of NJ and NL pigs. The results of multi omics joint analysis showed that betaine, uridine triphosphate, glycerol 3-phosphate, and glutathione in SDMs were enriched in the shared KEGG pathway and significantly correlated with C1QTNF12, GGA3, SLC16A6, and RXRG in DEGs. In general, it is feasible to enhance the production performance of NJ pigs through crossbreeding with LW pigs. The hybrid offspring inherit the advantages of these two varieties, maintaining excellent meat quality while also having better carcass performance.

## 1 Introduction

With the continuous development of science and technology and production methods, people’s living standards and consumption levels are constantly improving, and the requirements for pork quality are also increasing. Hence, the production of high-quality and safe pork is becoming increasingly urgent (1). Pork quality is influenced by many factors, including breed, nutrition, slaughter, and storage methods, with breed playing a crucial role (2). In the past, the breeding goal was to increase the meat yield and reduce the carcass fat. Large White pigs, Landrace pigs and other lean pig breeds with the advantages of fast growth and high feed efficiency were cultivated. However, this also led to a significant decline in the quality of pork. Unlike lean pig breeds, obese pig breeds have the characteristics of good meat quality, strong stress resistance, and resistance to roughage, especially with a higher intramuscular fat (IMF), which is favored by consumers.

According to the Domestic Animal Diversity in the World index, China has 118 different types of local pig breeds and abundant resources of obese pig breeds (3). Neijiang (NJ) pig is a typical obese local pig in China and is recognized as one of China’s top 10 famous pigs. Compared with the common commercial breeding pigs, NJ pigs have the advantages of stable genetic performance, good combining ability, strong adaptability, gentle temperament and resistance to roughage (4). However, NJ pigs have the drawbacks of slow growth rate and low meat production, and are in a weak position in the current pig market (5). Large White (LW) pigs have the advantages of high feed efficiency, high average daily gain and high lean meat rate, and have been widely used in the breeding industry (6). Heterosis is usually reflected in the hybridization process of two pure lines or inbred lines with different genetic composition (7). The offspring produced by hybridization usually exceed the parents in part or as a whole on a variety of different phenotypes. Since the beginning of the 20th century, heterosis has been discovered and applied to agricultural production, which has greatly promoted the improvement of animal and plant yield and quality (8–10).

Therefore, in order to improve the carcass performance of NJ pigs and give full play to the excellent resource characteristics of NJ meat quality, this study used heterosis to hybridize NJ pigs with LW pigs to cultivate NL pigs. We compared the differences in carcass and meat quality between NL pigs and NJ pigs, and speculated that these differences may be related to the composition of metabolites. To verify this hypothesis, we also conducted targeted metabolomics and transcriptomics analysis on NJ pigs for the first time, in order to explore the composition of muscle metabolites in NJ pigs and the differences in metabolites between NJ pigs and NL pigs, and to identify possible regulatory factors. The results of this study will provide valuable reference for the development and utilization of Chinese local pig germplasm resources and meat quality improvement.

## 2 Materials and methods

### 2.1 Ethics statement

All experimental procedures described below were approved by the Animal Ethical and Welfare Committee of Sichuan Agricultural University, Chengdu, China (Approval No. 2021302137, approval date: 1 July 2021).

### 2.2 Animals and samples preparation

In this study, the pigs were all from the same pig farm of a company in southwest China, including 5 NJ pigs and 5 NL pigs. All pigs were raised in the same breeding environment, adopted the same feeding mode, and fed the same batch of the same feed. Among them, NL pig is NJ pig as male parent and LW pig as female parent. When the feeding age reaches 180 days, the carcass and meat quality of NJ and NL pigs are measured. All pigs were slaughtered following the method of Du et al. (11). After transport to the abattoir, the pigs had no access to feed for 24 h before slaughter. The left longissimus dorsi muscle (LDM) were collected and snap-frozen in liquid nitrogen and stored at 80°C until further. Another of the LDM sample was put into paraformaldehyde solution for histological examination.

### 2.3 Carcass and meat quality measurement

Slaughter pigs were fasted for 24 h, free to drink water, and avoided fighting. Slaughter body weight was measured 2 h before slaughter. After the exsanguination of each pig, the hair was removed, the head, hoof, tail and internal organs were removed, and the kidney and plate oil were retained. The carcass was divided into two halves along the midline of the carcass, and the carcass weight was combined on both sides. The loin eye area (height × width × 0.7, cm^2^) and the average backfat thickness (mm) of the thickest part of the carcass shoulder, the last rib and the waist were measured with a vernier caliper (12). Subsequently, dissect the left side of the carcass, get all the lean meat, bones, fat and skin. The dressing percentage was calculated as the percentage of carcass weight to slaughter body weight, and the lean meat/fat rate was calculated as the percentage of lean meat/fat weight to carcass weight.

LDM samples for meat quality traits were collected from near the last rib on the left side of the carcass within 45 min after slaughter. The LDM samples (thickness of about 5 cm) from the first to the second thoracic vertebrae were taken, and the pH and meat color of the LDM were measured by pH meter and Minolta CR-300 colorimeter at 45 min after slaughter. Subsequently, the LDM samples were stored at 4°C, and the pH was measured again 24 h after slaughter. The drip loss, water-holding capacity and shear force of the LDM sample at the last rib were measured. Drip loss was measured by meat samples (approximately 30 g) storageat 4°C for 24 h, and the initial and final weights were calculated. The water-holding capacity was measured according to the method of Gan et al. (13). After slaughter, 100 g of LDM samples were taken and refrigerated for 48 h at 4°C. Three meat samples were taken using a standard sampler to measure the water-holding capacity and calculate the average value. Shear force (SF) was determined using a Texture Analyzer (TA.XT. Plus, Stable Micro Systems, Godalming, UK) equipped with a Warner-Bratzler shearing device.

### 2.4 Hematoxylin-eosin staining

After slaughter, the LDM samples at the 6th and 7th ribs on the left side of the carcass were quickly taken, fixed with paraformaldehyde and embedded in paraffin. The 5-μm sections were dewaxed and rehydrated and then stained with hematoxylin (Servicebio, Wuhan, China) solution for 3–5 min., Followed by staining with eosin (ServiceBio, Wuhan, China) for 3 min. The sections were dehydrated in 85% ethanol (Sangon, Shanghai, China) and 95% ethanol for 5 min. The sections were then dehydrated three times in 100% ethanol for 5 min each and sealed with neutral balsam (Sangon, Shanghai, China). ImageJ software was used to calculate the cross-sectional area of muscle fibers in the LDM samples, and at least three slices of each breed were counted to determine. The mean value was used for statistical analysis.

### 2.5 Transcriptome data analysis

The RNA seq platform is Illumina Novaseq 6000, and the sequencing service is provided by Novogene Co., Ltd. (Beijing, China). The measurement data is monitored for quality using Fastp software and Clean reads are obtained. The RNA seq platform is Illumina Novaseq 6000, and the sequencing service is provided by Novogene Co., Ltd. (Beijing, China). The measurement data is monitored for quality using Fastp software and Clean reads are obtained. The clean data were aligned to the reference genome (Sscrofa11.1) using hisat2 (v2.2.1). Subsequently, RNA seq quantification was performed using Kallisto (0.44.0) and standardized using the TPM algorithm. After obtaining differential gene data, principal component analysis (PCA) analysis and partial least squares discriminant analysis (PLS-DA) analysis were performed using the online OmicShare tools (http://www.omicshare.com/tools, last accessed on 25 Aug 2024). Clustering Heatmap and volcano plot was plotted by https://www.bioinformatics.com.cn (last accessed on 25 Aug 2024), an online platform for data analysis and visualization. Use the DESeq2_1.44 R package (R, version “4.4”) to screen for differentially expressed genes (| log2FC | > 1, *p* value<0.05), and perform GO enrichment analysis and KEGG pathway enrichment analysis on the selected differentially expressed genes annotation using OmicShare tools (https://www.omicshare.com/tools, last accessed on 25 Aug 2024).

### 2.6 Targeted metabolome analysis

Subsequently, the samples were analyzed by liquid chromatography-mass spectrometry (LC–MS) of BioProfle Biotechnology (Shanghai, China). Specifically, Shimadzu Nexera X2 LC-30 AD system equipped with ACQUITY UPLC HSS T3 chromatographic column (1.8 μm, 2.1 × 50 mm chromatographic column, Waters) and triple quadrupole mass spectrometer (5500 QTRAP, AB SCIEX). Metabolites were detected in electrospray negative ionization and positive ionization modes. The mass spectrometer conditions are set as follows: negative ionization: source temperature 550°C, ion source gas 1 (GAS1): 40, ion source gas 2 (GAS2):50, curtain gas (CUR): 35, ion spray voltage fluctuation (ISVF): −4,500 V; positive ionization: source temperature 550°C, ion source gas 1 (GAS1): 40, ion source gas 2 (GAS2): 50, curtain gas (CUR): 35, ion spray voltage floating (ISVF): 5,500 V. MRM (Multiple Reaction Monitoring) mode was used to detect the transition. The original MRM data for the MT1000 KIT metabolites were extracted using MultiQuant 3.0.2 software, and the peak areas for each metabolite were subsequently obtained. Discriminant metabolites were obtained using the statistically significant threshold of the variable-to-projection (VIP) value obtained from the PLS-DA model and the two-tailed student *t*-test (*p*-value) of the standardized raw data. Metabolites with VIP greater than 1 and *p* value less than 0.05 were considered as statistically significant metabolites. Cluster heatmap and volcano map was plotted by https://www.bioinformatics.com.cn. The KEGG database was used to analyze the enrichment pathways of the changed metabolites (http://www.omicshare.com/tools, last accessed on 25 Aug 2024). The experimental method is from Shanghai Bioprofile.

### 2.7 Statistical analyses

In the phenotypic data analysis stage of this study, the data entry and preliminary collation were completed by Excel spreadsheet tool. Difference analysis and correlation analysis were performed using SPSS 22.0 (IBM, United States). In the statistical analysis results, when the *p* value was less than 0.05, the difference was considered significant. In the correlation analysis, the absolute value of the Pearson correlation coefficient greater than 0.8 indicates a high degree of strong correlation, 0.5–0.8 indicates a strong correlation, 0.3–0.5 indicates a moderate intensity correlation, and less than 0.3 is considered as a weak correlation.

## 3 Results

### 3.1 Carcass characteristics

**Figure 1:**
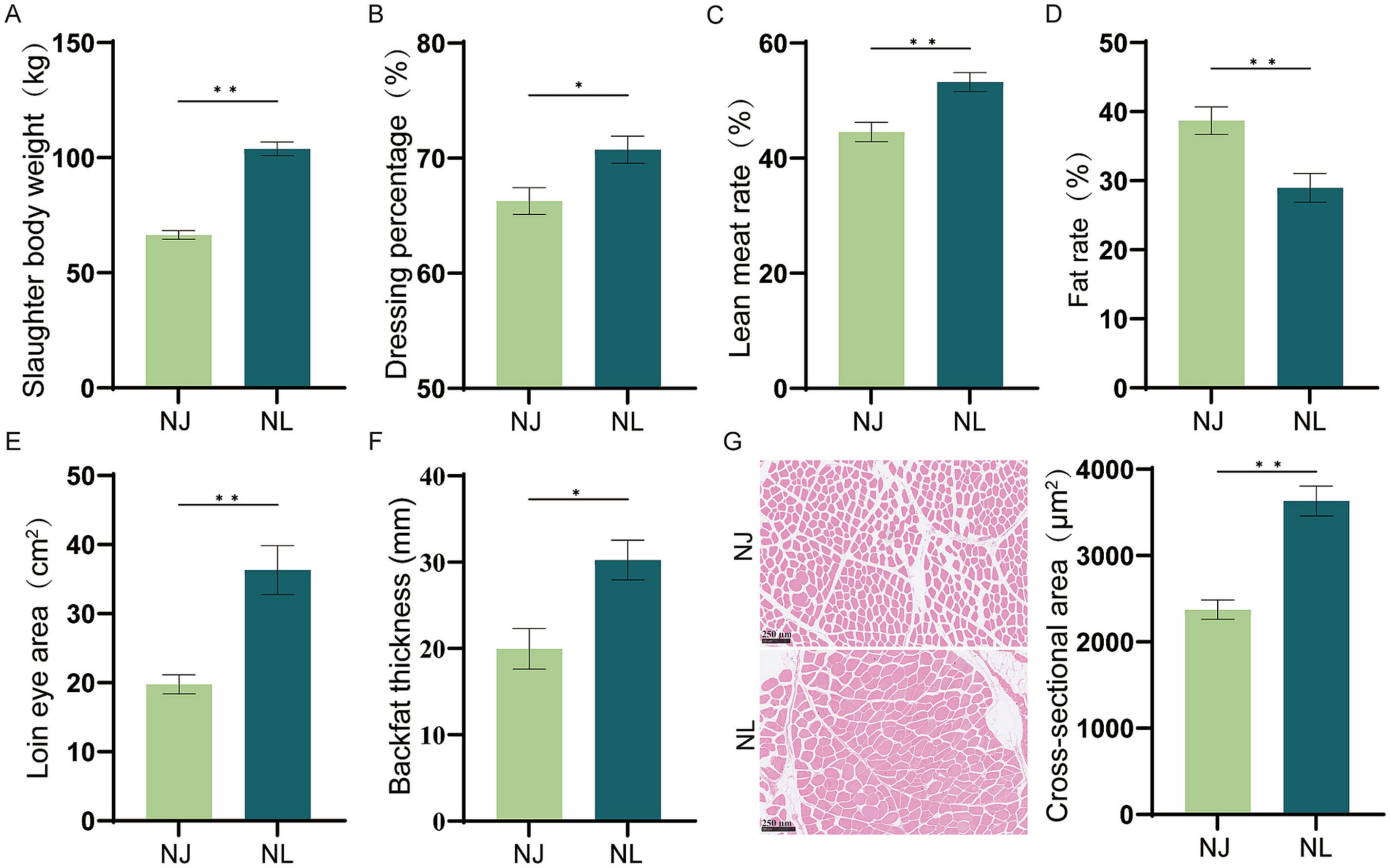
Comparative analysis of carcass traits between NJ and NL pigs. (A) Slaughter body weight. (B) Dressing percentage. (C) Lean meat rate. (D) Fat rate. (E) Loin eye area of pigs. (F) Average backfat thickness. (G) Hematoxylin–eosin staining and mean muscle fibers cross-sectional area of longissimus thoracis muscle. Data are expressed as the mean ± SEM. *p < 0.05, **p < 0.01.

The comparison results of carcass traits between NJ pig and NL pig are shown in Figure 1, including the slaughter body weight, dressing percentage, lean percentage, fat percentage, loin eye area, backfat depth, loin eye area, differed significantly between the NJ pigs and the NL pigs. Meanwhile, slaughter body weight, dressing percentage, lean meat rate, loin eye area, backfat thickness and cross-sectional area of NJ pig were significantly higher than NL pig, and fat rate of NJ pigs were significantly lower than NL pigs.

### 3.2 Meat quality trait

**Figure 2:**
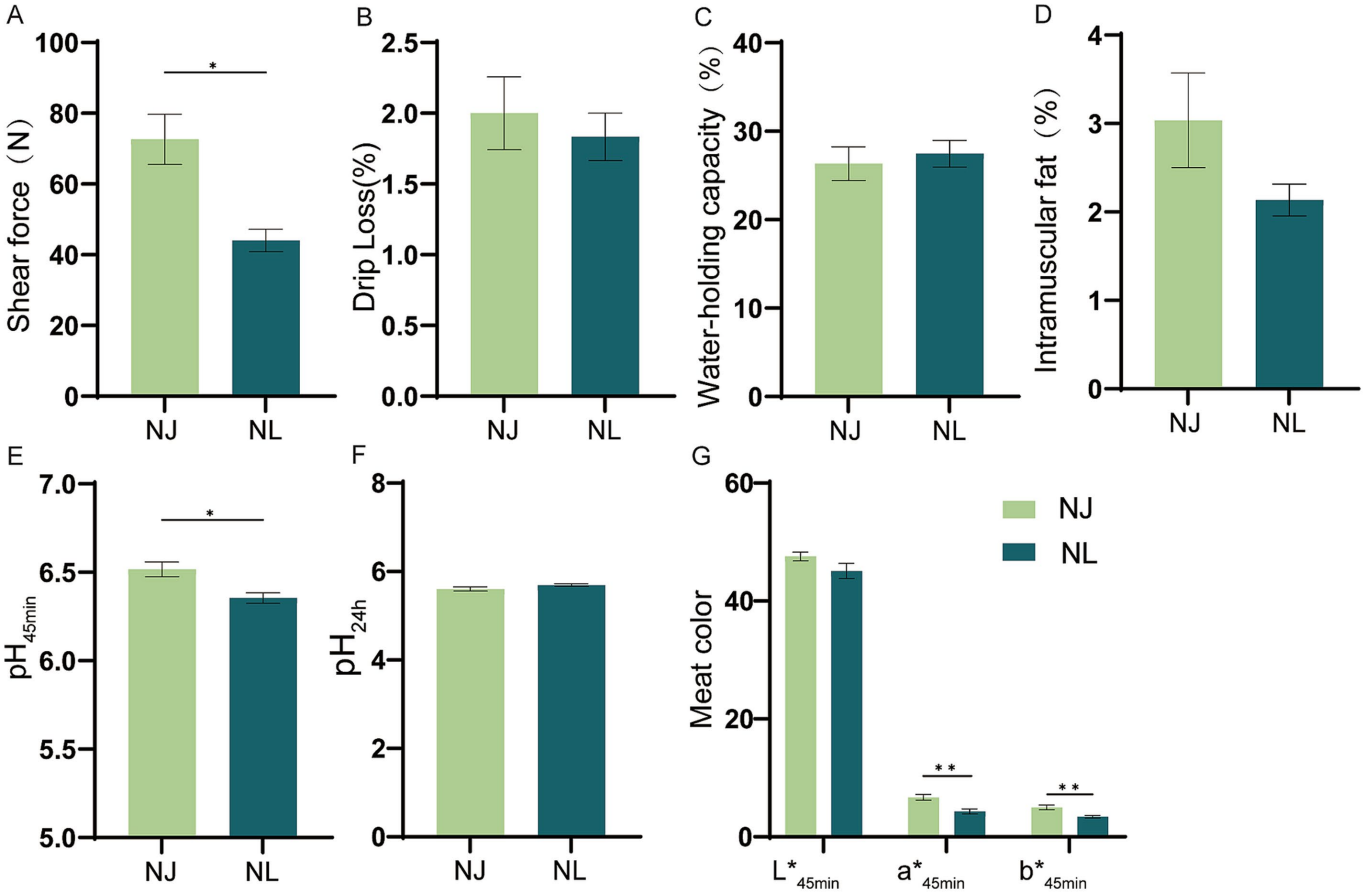
Meat quality of NJ and NL pigs. (A) Shear force. (B) Drip loss. (C) Water-holding capacity. (D) Intramuscular fat content. (E) pH_45min._ (F) pH_24min_. (G) CIE LAB color values of meat at 45 min after slaughter. Data are expressed as the mean ± SEM. *p < 0.05, **p < 0.01.

As shown in Figure 2, the shear force, pH_45min_, meat redness (a*_45min_) and meat yellowness (b*_45min_) of the LDM in NJ pigs were significantly higher than NL pigs. However, there were no difference in the drip loss, water-holding capacity, intramuscular fat, pH_24h_ and meat lightness (L*_45min_) between NJ pigs and NL pigs in the LDM.

### 3.3 Amino acid and fatty acid metabolic analysis

The composition of amino acids and fatty acids in the LDM was identified using targeted metabolomics methods. In our study, there were no significant differences in the amino acid composition of the LDM between NJ and NL pigs in terms of TAA, EAA, and NEAA (Table 1). Further analysis found that among the eight EAA and ten NEAA detected, only the level of Asn in NL pigs were significantly lower than those in NJ pigs.

**Table 1:** The amino acid content of the LDM in NJ and NL pigs (peak area/10,000).

Items	NJ	NL	FC	*p* value
EAA				
Lys	1520.22 ± 273.39	978.88 ± 581.48	0.64	0.10
Ile	2484.52 ± 452.34	1912.85 ± 369.05	0.77	0.06
Leu	2942.08 ± 902.34	2507.19 ± 226.68	0.85	0.33
Val	1710.48 ± 276.50	1606.05 ± 107.55	0.94	0.45
Thr	12.11 ± 3.41	10.38 ± 3.90	0.86	0.48
Phe	3750.28 ± 630.67	3839.03 ± 350.41	1.02	0.79
Met	245.96 ± 39.49	292.46 ± 69.76	1.19	0.23
Trp	2311.82 ± 428.71	2728.97 ± 187.56	1.18	0.08
NEAA				
His	2076.24 ± 218.92	2285.33 ± 162.77	1.10	0.12
Gln	1175.85 ± 254.91	739.20 ± 431.87	0.63	0.09
Arg	96.90 ± 15.80	92.25 ± 22.03	0.95	0.71
Glu	90.36 ± 77.09	51.04 ± 26.90	0.56	0.31
Ser	24.58 ± 8.96	37.59 ± 19.23	1.53	0.21
Gly	1.93 ± 1.28	2.02 ± 0.69	1.04	0.90
Tyr	770.20 ± 126.02	816.84 ± 116.33	1.06	0.56
Ala	282.41 ± 52.15	233.64 ± 45.57	0.83	0.15
Asn	1.82 ± 0.49	1.02 ± 0.59	0.56	0.05
Pro	141.80 ± 29.41	138.32 ± 25.74	0.98	0.85
EAA	14977.48 ± 2088.74	13875.81 ± 613.70	0.93	0.29
NEAA	4662.11 ± 310.86	4397.27 ± 342.50	0.94	0.24
TAA	19639.59 ± 2359.46	18273.08 ± 725.97	0.93	0.25

According to the fatty acid spectrum shown in Table 2, we found no significant differences in ∑SFA, ∑UFA, ∑MUFA, ∑PUFA, ∑n3, ∑n6, n6/n3, and TFA. Similarly, we did not observe any individual fatty acids with significant differences between the LDM of NJ and NL pigs.

**Table 2:** The fatty acid content in the LDM of NJ and NL pigs (peak area/10,000).

Items	NJ	NL	FC	*p* value
C5:0	37.35 ± 26.95	34.36 ± 19.13	0.92	0.84
C6:0	12.40 ± 2.84	9.36 ± 3.98	0.75	0.20
C10:0	11.01 ± 1.20	11.81 ± 2.08	1.07	0.48
C12:0	241.98 ± 85.88	234.05 ± 51.87	0.97	0.86
C14:0	195.27 ± 51.44	184.46 ± 16.12	0.94	0.67
C15:0	496.45 ± 348.08	526.11 ± 82.87	1.06	0.86
C16:0	3873.80 ± 537.33	3765.09 ± 644.52	0.97	0.78
C16:1	1097.12 ± 426.96	1062.50 ± 120.82	0.97	0.87
C16:2	365.74 ± 15.86	368.65 ± 20.36	1.01	0.81
C17:0	39.85 ± 27.07	34.49 ± 4.75	0.87	0.67
C18:0	88.95 ± 34.11	76.62 ± 23.84	0.86	0.53
C18:1	3323.60 ± 994.22	3163.17 ± 746.78	0.95	0.78
C18:2	2405.32 ± 549.44	2390.05 ± 452.66	0.99	0.96
C18:3	299.16 ± 126.59	307.80 ± 157.87	1.03	0.93
C20:0	3316.47 ± 1127.10	3821.64 ± 516.08	1.15	0.39
C20:1	1286.32 ± 978.48	613.50 ± 121.82	0.48	0.17
C20:2	201.86 ± 32.54	216.56 ± 48.21	1.07	0.59
C20:3	240.79 ± 71.55	247.20 ± 55.83	1.03	0.88
C20:4	918.81 ± 182.16	985.90 ± 111.17	1.07	0.50
C22:0	231.77 ± 78.74	246.05 ± 31.44	1.06	0.72
C22:1	116.01 ± 70.64	48.03 ± 11.69	0.41	0.07
C22:2	11.95 ± 9.49	11.56 ± 3.11	0.97	0.93
C22:3	6.53 ± 3.17	6.66 ± 2.85	1.02	0.95
C22:4	217.49 ± 125.95	248.62 ± 78.27	1.14	0.65
Eicosapentaenoic acid	5.93 ± 1.19	7.00 ± 1.12	1.18	0.18
Hydroxyisocaproic acid	1429.62 ± 485.81	1299.86 ± 233.38	0.91	0.60
Sebacic acid	125.63 ± 21.09	113.78 ± 18.68	0.91	0.37
Tridecanoic acid	54.58 ± 12.48	51.46 ± 29.81	0.94	0.83
13S-hydroxyoctadecadienoic acid	46.81 ± 24.40	36.89 ± 14.89	0.79	0.46
Avocadyne 1-acetate	6.60 ± 3.45	6.43 ± 2.50	0.97	0.93
beta-Hydroxymyristic acid	10.64 ± 5.61	8.45 ± 3.23	0.79	0.47
ΣSFA	8736.14 ± 2108.56	9117.74 ± 1099.34	1.04	0.73
ΣUFA	11979.66 ± 2307.69	11020.40 ± 1563.14	0.92	0.46
ΣMUFA	5829.65 ± 1306.12	4893.63 ± 945.02	0.84	0.23
ΣPUFA	6150.01 ± 1134.92	6126.77 ± 725.04	1.00	0.97
Σn3	305.09 ± 127.65	314.80 ± 158.51	1.03	0.92
Σn6	2669.62 ± 652.52	2675.57 ± 532.54	1.00	0.99
n 6/ n 3	9.53 ± 2.72	9.28 ± 2.10	0.97	0.87
TFA	20715.81 ± 4269.07	20138.13 ± 2539.24	0.97	0.80

ΣSFA, sum of saturated fatty acids; ΣUFA, sum of unsaturated fatty acids; ΣMUFA, sum of monounsaturated fatty acids; ΣPUFA, sum of polyunsaturated fatty acids; TFA, total fatty acids.

### 3.4 Transcriptome analysis of the LDM in NJ and NL pigs

**Figure 3:**
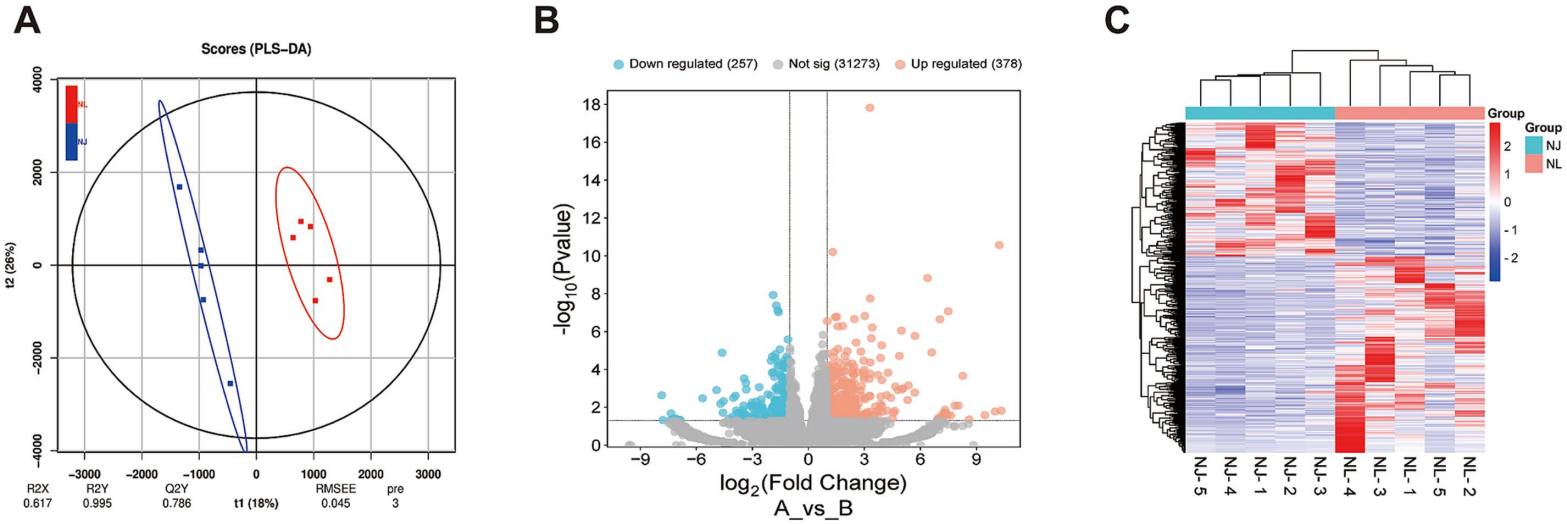
Differentially expressed genes (DEGs) the LDM of NJ and NL pigs. (A) Partial Least Squares Discrimination Analysis (PLS-DA) score plot. (B) Volcano plot of identified genes including up-regulated and down-regulated genes in the RNA-seq. (C) Heat map of DEGs.

To investigate the phenotypic differences between hybrid pigs and NJ pigs at the molecular level, this study sampled the LDM from slaughtered pigs and conducted transcriptome sequencing analysis. The results of partial least squares discriminant analysis (PLS-DA) showed that there were differences in gene expression between NJ pigs and NL pigs (Figure 3A). We used the R package DESeq2 to screen for DEGs, using FC >2 or FC < 0.5 and *p*-value < 0.05 as screening criteria, and identified 635 DEGs, of which 378 DEGs were upregulated and 257 DEGs were downregulated in NL pigs (Figure 3B). The clustering analysis heatmap of DEGs shows that gene expression patterns are clustered within groups, with significant differences between groups (Figure 3C). In summary, we found significant differential expression of genes between NJ pigs and NL pigs in the LDM tissue.

**Figure 4:**
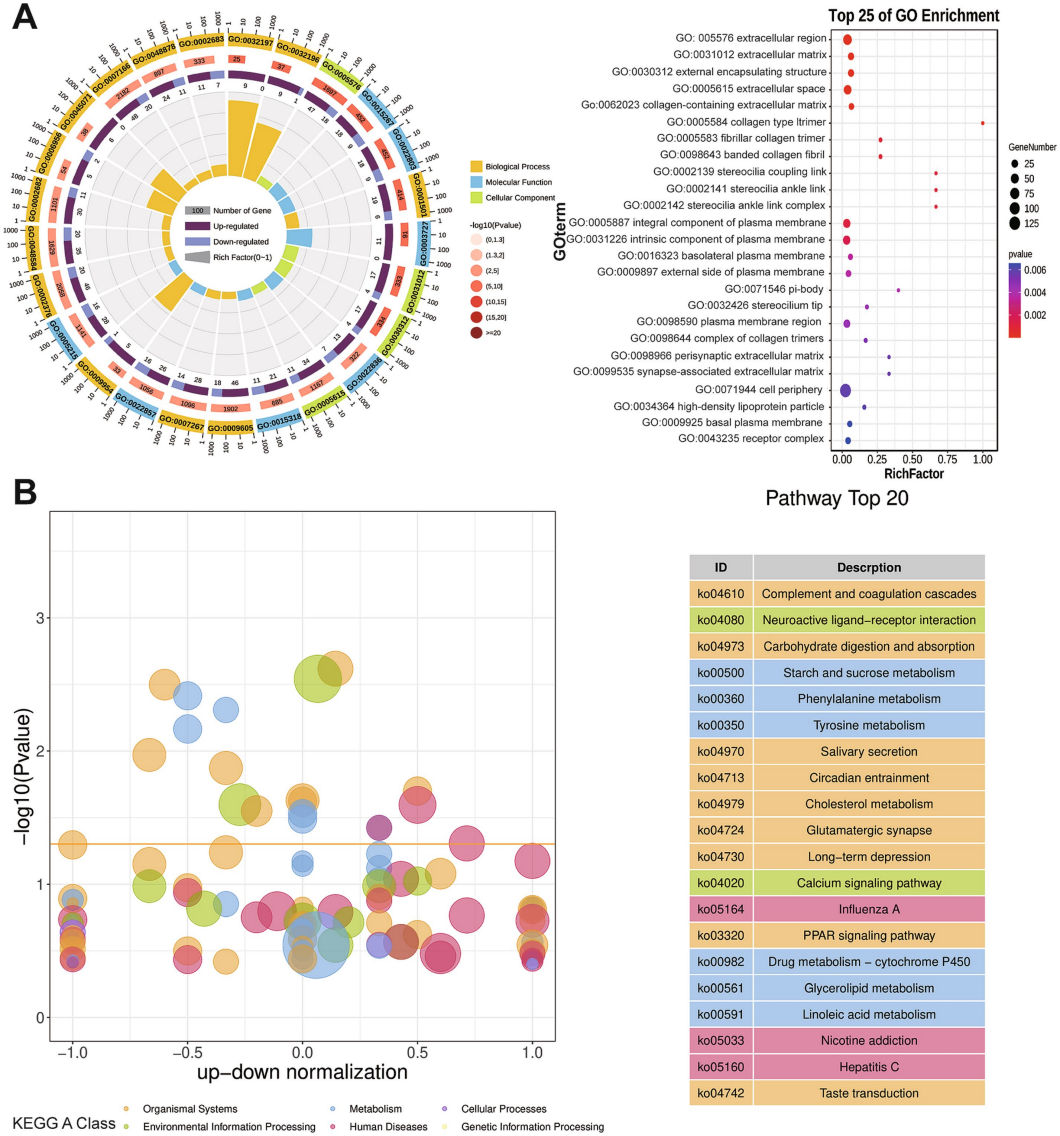
GO and KEGG enrichment analysis of DEGs between NJ and NL pigs LDM. (A) The Gene Ontology (GO) enrichment analysis results. (B) Highlight the bubble plot of KEGG pathway enrichment differences. There is a yellow line of 0.05 at the *Q*-value threshold.

Gene Ontology (GO) enrichment analysis of DEGs between NJ pigs and NL pigs was conducted, with particular focus on molecular function, cellular component, and biological process categories (Figure 4A). The DEGs between NJ and NL pigs were primarily enriched in the biological process ontology terms skeletal system development and cell–cell signaling. The cellular component ontology terms with the highest gene counts were extracellular region and extracellular matrix, while the molecular function ontology terms with the highest gene counts were transmembrane transporter and channel activity. To further investigate the function of DEGs, we used KEGG enrichment analysis. Specifically, 20 pathways exhibited significant changes in NL pigs compared to NJ pigs, including carbohydrate digestion and absorption, phenylalanine metabolism, tyrosine metabolism, and starch and glycerolipid metabolism (Figure 4B).

### 3.5 Targeted metabolomic analysis of the LDM in NJ and NL pigs

**Figure 5:**
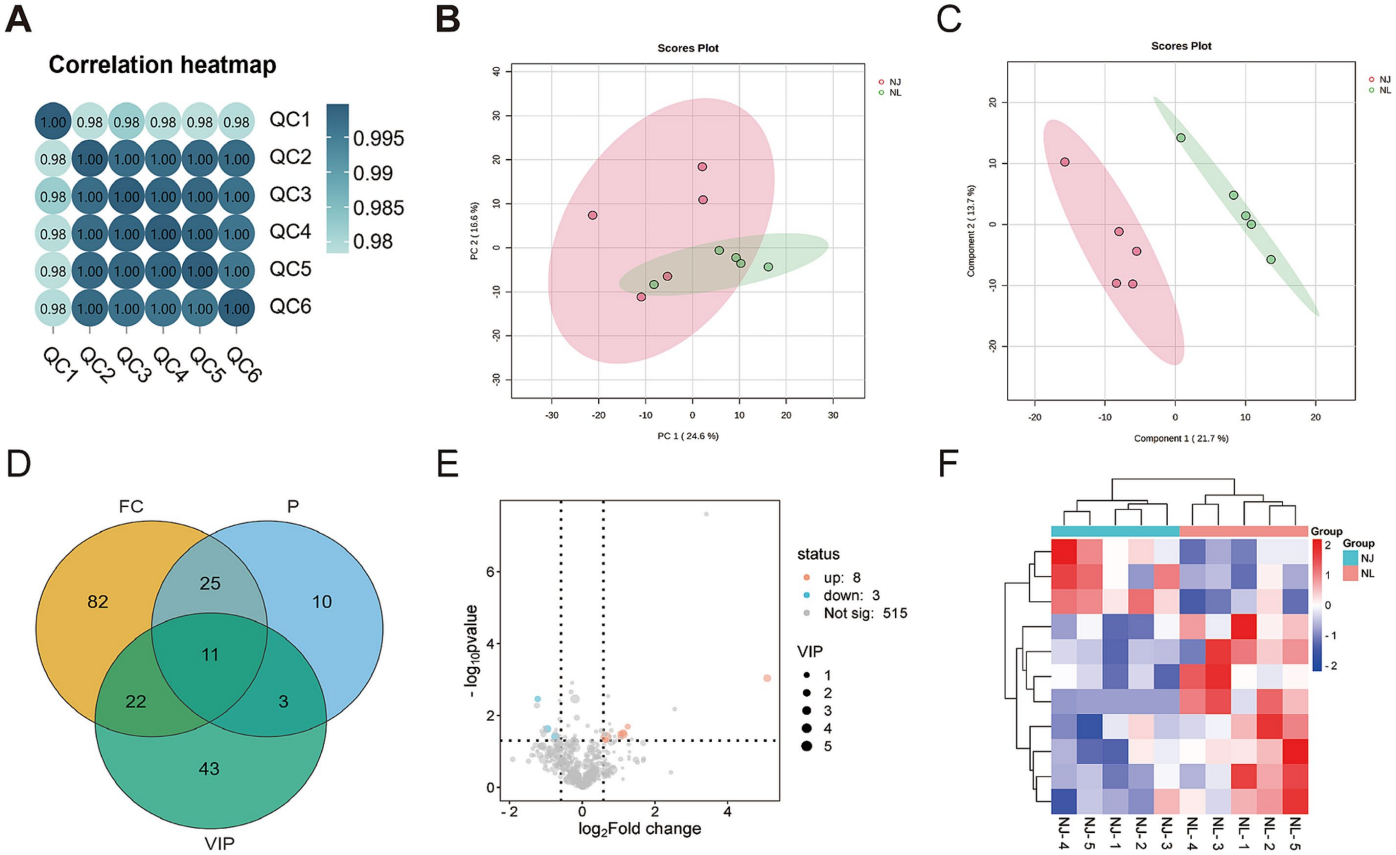
Multivariate analysis of LDM metabolites in NJ and NL pigs. (A) Quality control analysis. (B) Principal component analysis. (C) Partial Least Squares Discrimination Analysis (PLS-DA) score plot. (D) Wayne diagram of three metabolite screening methods. (E) Volcanic plot. (F) Cluster heatmap of important differential metabolites.

**Figure 6:**
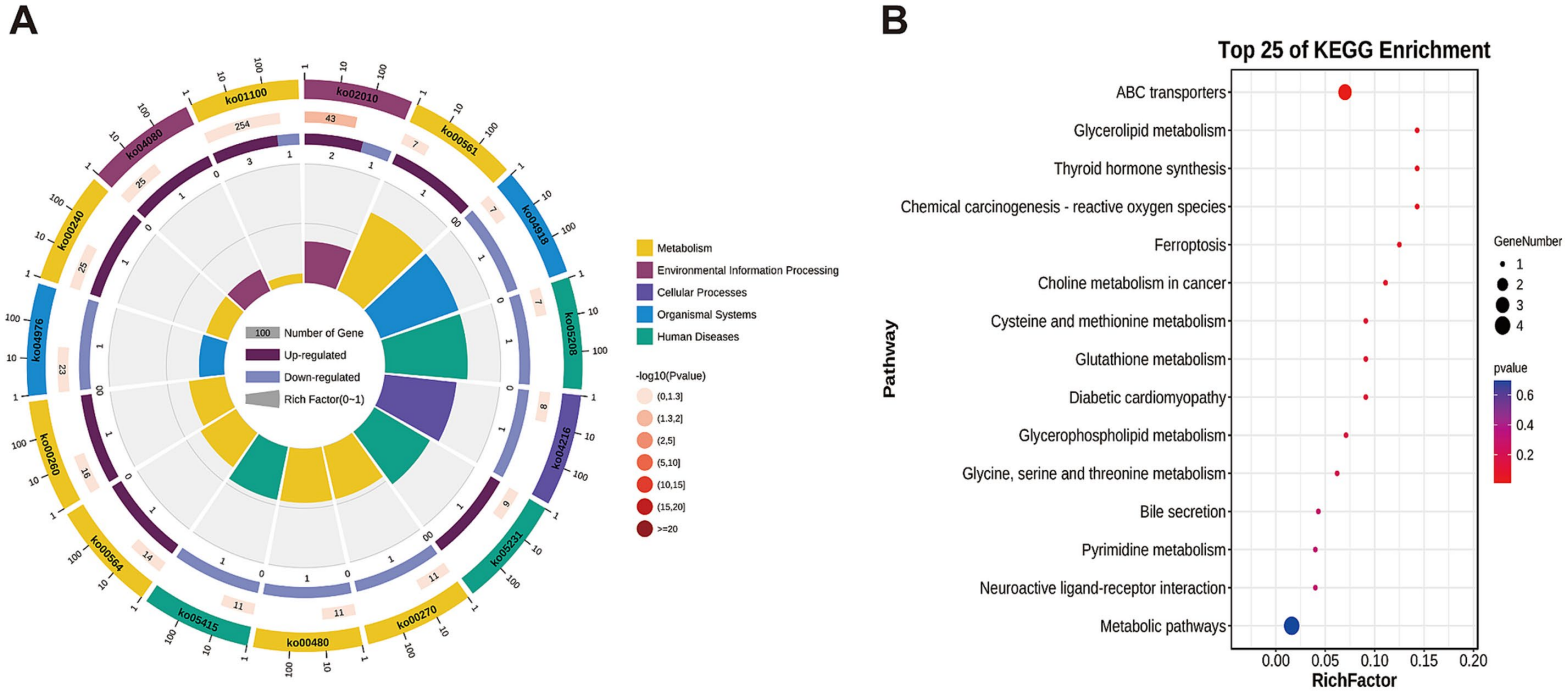
KEGG enrichment results for identified SDMs. (A) DAM-related pathway distributions. (B) KEGG enrichment analysis results for SDMs, with the pathway name shown along the y-axis and the degree of enrichment along the x-axis.

In this study, targeted metabolomics was used to determine the muscle metabolites of NJ pigs and NL pigs to detect the overall biochemical changes. In the analysis of quality control samples, the correlation of all six quality control samples exceeded 0.98, indicating that the detection process was stable and reliable (Figure 5A). Through LC/MS metabolite analysis, a total of 526 metabolites were found in all samples. The PCA results showed that there was no significant difference in the metabolome of LDM between NJ pigs and NL pigs (Figure 5B). Further analysis using PLS-DA revealed significant differences in the metabolic profiles of LDM between NJ and NL pigs (Figure 5C). According to the criteria of FC >1.5 or FC < 0.67, *p* < 0.05, and PLS-DA VIP > 1, a total of 11 significantly different metabolites (SDMs) were identified in the LDM of NJ and NL pigs, with 8 SDMs upregulated and 3 SDMs downregulated (Figure 5D). These metabolites were described in the volcano map (Figure 5E) and heatmap (Figure 5F) for visual comparison. The three metabolites with the highest upregulation are PS (18:0/18:2), uridine triphosphate, and 3-phosphoglycerate, while the three metabolites with the highest downregulation include LPI (18:2), 3-hydroxyhexanoyl carnitine, and glutathione. In addition, KEGG enriched pathways showed that these SDMs were significantly enriched in glycerolipid metabolism, glutathione metabolism, glycerophoripid metabolism, and metabolic pathways (Figures 6A,B).

### 3.6 Combined analysis of transcriptome and metabolomics

**Figure 7:**
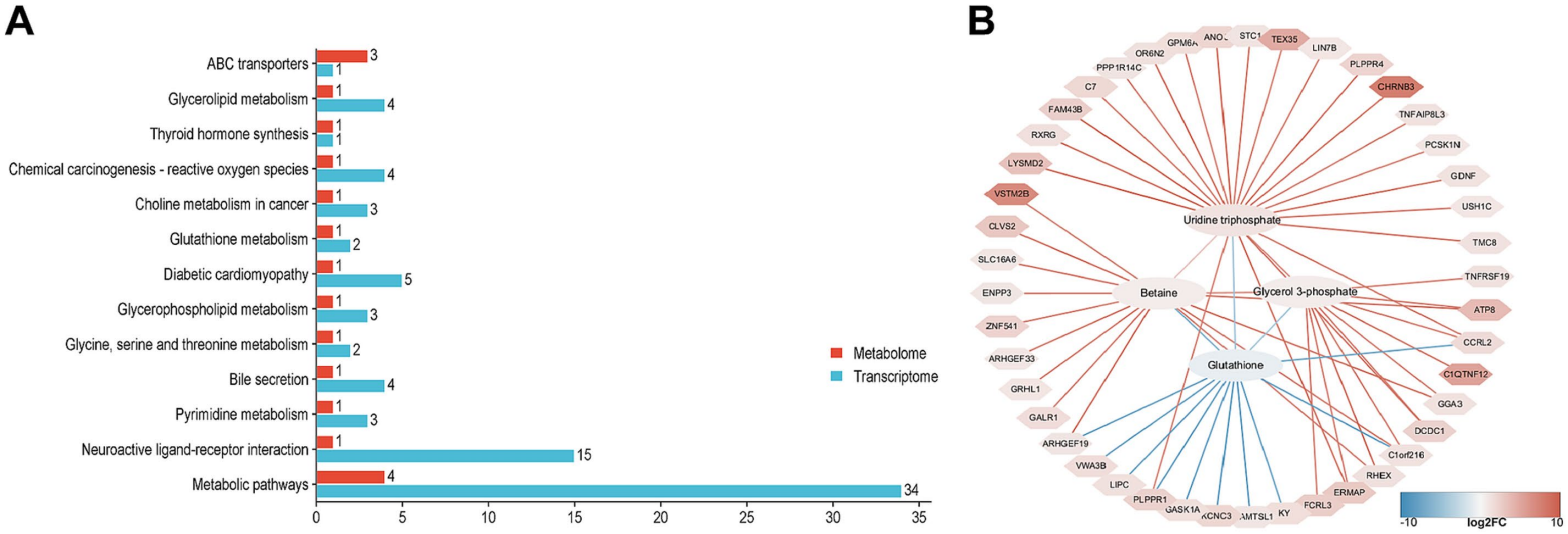
Correlation analysis between transcriptomics and metabolomics. (A) KEGG pathway enrichment of DEGs and SDMs. (B) Correlation network diagram, ellipses are the names of SDMs, polygons are the names of DEGs, red lines represent positive correlations, and blue lines represent negative correlations.

Based on KEGG pathway enrichment analysis, enrich all DEGs and SDMs to determine the pathways associated with DEGs and SDMs. DEGs and SDMs are significantly enriched in 13 KEGG pathways, including glycerolipid metabolism, glutathione metabolism, glycerophospholipid metabolism, glycine, serine and threonine metabolism, pyridine metabolism, and metabolic pathways. Further analysis shows that all 13 KEGG pathways are associated with 4 SDMs, including glutathione, uridine triphosphate, glycerol 3-phosphate, and betaine. To investigate the relationship between DEGs and SDM in NJ and NL pigs, we utilized the Pearson algorithm to construct a regulatory network between genes and metabolites. The results showed that glycerol 3-phosphate was associated with C1QTNF12 and GGA3, glutathione was associated with CCRL2, Betaine and SLC16A6, Uridine triphosphate and RXRG (| PCC | > 0.5, *p* < 0.05) (Figure 7).

## 4 Discussion

In order to meet health needs and pursue taste and flavor, consumers pay close attention to the quality and nutritional value of meat represented by pork. Previous studies have shown that Chinese indigenous pig breeds have better meat quality, better fatty acid and amino acid composition, and are more suitable for human consumption (14). NJ pig is a local pig breed in Sichuan Basin of China, which is famous for its delicious meat and strong stress resistance. However, local breeds such as NJ pigs also exhibit issues such as low feed conversion efficiency, and low lean meat percentage (15). Therefore, this study aims to utilize heterosis to improve NJ pigs by crossbreeding with LW pigs. The present study compared the carcass and meat quality traits of NJ pigs with their hybrid offspring NL pigs, and for the first time analyzed potential regulatory mechanisms using transcriptomics and metabolomics.

The carcass quality of pigs is determined by the proportion and distribution of muscle, sebum and bone. According to the current market situation and consumer demand in China, slaughter rate and lean meat rate are undoubtedly the most concerned carcass traits (16). The lean meat rate of lean-type pigs such as DLY pigs can exceed 60%, whereas certain local pig breeds typically exhibit a lean meat rate of around 40% (17, 18). Hybrid commercial pigs, resulting from the crossbreeding of these lean-type pigs with local breeds, usually have a lean meat rate of approximately 55% (19, 20). In this study, the lean meat rate of NL pigs at the same age was 53.17 ± 3.92%, significantly higher than that of NJ pigs at 44.55 ± 3.72%. This indicates that the crossbreeding strategy effectively improves the meat production capacity of NJ pigs, aligning with the characteristics of both local and hybrid pigs. The results of slaughter rate and loin eye area measurements further support this finding. On the other hand, the NL pigs not only had a larger loin eye area and higher lean meat rate compared to the NJ pigs, but also a lower backfat thickness. These findings are consistent with previous research, further confirming the highly significant positive correlation between loin eye area and lean meat rate (21, 22). This correlation suggests that loin eye area can be effectively used as an auxiliary trait for selecting pigs with higher carcass lean meat rate. Additionally, slaughter body weight is a major factor affecting pork yield. The study results indicated that NL pigs of the same age had significantly higher slaughter body weight compared to NJ pigs, suggesting that NL pigs have faster growth rate. Based on the above results, compared with NJ pigs, the carcass traits of hybrid offspring NL pigs have been significantly improved.

Whether the hybrid offspring of NJ pigs can inherit the excellent meat quality traits of NJ pigs is also an important criterion for evaluating the effectiveness of hybrid improvement. Meat quality is a crucial factor influencing consumer choice, which in turn affects the profitability of the meat industry (23). At the same time, meat quality is a complex trait affected by many factors, which can be divided into sensory quality and nutritional quality (24–26). Sensory quality mainly encompasses characteristics such as pH, meat color, water-holding capacity, and shear force, which directly affect consumer perception (27). The pH of pork at 45 min post-slaughter can directly reflect the initial acidity of the meat, significantly affecting its water-holding capacity, tenderness, and the preliminary identification of inferior meat (28). In this experiment, the pH_45min_ levels of both NJ pigs and NL pigs were within the normal range, with no occurrence of inferior meat. Meat color is influenced by factors such as myoglobin content, residual hemoglobin content, and muscle hemoglobin content (29). In this study, there was no significant difference in L*_45min_ between the LDM of NJ pigs and NL pigs, but the a*_45min_ and b*_45min_ of NL pigs showed a significant decrease. The results also showed that the shear force of the LDM in NL pigs was significantly lower than that in NJ pigs, and the cross-sectional area was significantly higher than that in NJ pigs. This result supports the negative correlation between pork muscle fiber size and tenderness (30, 31). Pig carcass quality and lean meat percentage are directly influenced by subcutaneous fat deposition. Increasing the backfat thickness of finishing pigs can enhance IMF content, flavor, and juiciness of the LDM, thereby improving pork quality (32). Compared to lean-type three-way crossbred pigs, Chinese local pig breeds are characterized by higher IMF content (33). Our data shows that there is no significant difference in the IMF content of the LDM between NL pigs and NJ pigs. Compared to previously reported results, the IMF levels of NL pigs are significantly higher than those of LW pigs (34, 35). In general, the meat quality of NL pigs is still at a good level, which belongs to high-quality pork.

In recent years, RNA seq sequencing technology has been widely used to explore genes related to important economic traits in pigs. In order to investigate the underlying causes of phenotypic differences between NJ pigs and hybrid pigs, this study conducted transcriptomic measurements on the LDM of NJ pigs and hybrid pigs. In this study, we identified a total of 635 DEGs in NJ and NL pigs, of which 378 DEGs were upregulated and 257 DEGs were downregulated. Among these DEGs, we identified differentially expressed genes that may affect the meat production performance of NJ pigs, including C1QTNF12 (log_2_ FC = 5.36), GGA3 (log_2_ FC = 1.35), SLC16A6 (log_2_ FC = 1.05), and RXRG (log_2_ FC = 1.44). The C1QTNF12 gene is predicted to have hormone activity and participate in various biological processes, including negative regulation of gluconeogenesis, positive regulation of glucose uptake, and lipid metabolism (36, 37). GGA3 is a gene encoding a gamma adaptive protein located in the Golgi apparatus and a member of the ARF binding (GGA) family. Studies have found that GGA3 is involved in glucose level regulation (38). SLC16A6 is a gene belonging to the monocarboxylate transporter family, and studies have found that this gene may regulate height by modulating the transport of monocarboxylic acids in multiple tissues (39). The RXRG gene is a member of the nuclear receptor class retinol X receptor (RXR) family. Studies have found that this gene is involved in the PPARA signaling pathway and adipocyte cytokine signaling pathway, and may play a key role in regulating IMF deposition (40). Someone has suggested that this gene has a potential relationship with muscle development and can serve as a biomarker for muscle atrophy (41). In our study, we found that the expression of the three genes mentioned above was significantly higher in NL pigs than in NJ pigs, which may increase the muscle content of NL pigs by regulating energy metabolism and muscle development.

Metabolites are the end products of gene expression and can reflect the actual biochemical changes and physiological states occurring within an organism (42). These changes directly influence the organism’s phenotypic traits. Therefore, there is a close relationship between muscle metabolites and meat quality. To investigate the changes in metabolites between NJ pigs and NL pigs, LC/MS was used to identify the composition and content of metabolites, and to analyze the metabolite profiles of the LDM from both pig breeds. We first analyzed the muscle amino acid and fatty acid composition in NJ pigs and NL pigs, and found no significant differences between the two groups. In this study, we detected 526 metabolites, including 11 SDMs, in the LDM of NJ and NL pigs. Betaine (log_2_ FC = 0.64) and Uridine triphosphate (UTP) (log_2_ FC = 1.25) in the LDM of NL pigs were significantly upregulated compared to NJ pigs, while Glutathione (GSH) (log_2_ FC = −0.74) was significantly downregulated. Betaine is an organic compound that exists in various organisms and plays multiple roles in them. Long term feeding of betaine can selectively increase the IMF content in pigs (43), and there are also reports that feeding betaine can increase pig carcass weight, loin eye area, and lean meat percentage (44). UTP is a nucleotide that plays an important role in RNA synthesis, glucose metabolism, cell signal transduction, glycoprotein and glycolipid synthesis and nucleotide metabolism (45–47). In the glucose metabolism pathway, UTP reacts with glucose-1-phosphate to produce UDP-glucose, which is then converted into glycogen for energy storage (48). Recent studies have also found that UTP is also involved in adipogenesis, adipocyte differentiation and pyruvate decomposition (49). Glycerol 3-phosphate is an important metabolic intermediate involved in lipid synthesis, glycolysis, and gluconeogenesis (50, 51). GSH is a molecule composed of glutamic acid, cysteine, and glycine, involved in protein synthesis and repair (52). Therefore, from a metabolomics perspective, this explains the physiological reasons why NL pigs have superior growth and development capabilities compared to NJ pigs. Further KEGG enrichment analysis of differential metabolites showed that the differential metabolites between NJ pigs and NL pigs were mainly concentrated in metabolic pathways, including glycolipid metabolism, glycine, serine and threonine metabolism and pyrimidine metabolism. Among them, glycolipid metabolism is one of the important signaling pathways in organisms, mainly involving the synthesis, transportation, decomposition, and utilization of triglycerides. It is crucial for maintaining the structural integrity of cell membranes, regulating cellular energy metabolism, and participating in various physiological functions (53). Therefore, the metabolic differences between NJ pigs and NL pigs may be a potential reason for the differences in carcass performance between the two groups, but further research is needed to determine the nature and mechanism of this relationship. Overall, the differences in metabolite composition between NL pigs and NJ pigs were relatively small.

## 5 Conclusion

In summary, this study shows that by crossbreeding NJ pigs and LW pigs, the hybrid offspring NL pigs can not only have better carcass performance than NJ pigs, but also inherit the excellent meat quality characteristics of NJ pigs. Through comprehensive transcriptomic and metabolomic analysis, we found that C1QTNF12, GGA3, SLC16A6, and RXRG genes are closely related to SDMs in metabolic pathways and play important roles in muscle development and metabolic processes in NL pigs. This study provides a reference for the development and utilization of local pig breeds in China and the improvement of meat quality of commercial pigs.

## Data Availability

The data presented in the study is stored in the Genome Sequence Archive repository with access number CRA020346.
